# Carbon transportation, transformation, and sedimentation processes at the land-river-estuary continuum

**DOI:** 10.1016/j.fmre.2022.07.007

**Published:** 2022-07-28

**Authors:** Yang Gao, Junjie Jia, Yao Lu, Kun Sun, Jing Wang, Shuoyue Wang

**Affiliations:** a Key Laboratory of Ecosystem Network Observation and Modeling, Institute of Geographic Sciences and Natural Resources Research, Chinese Academy of Sciences, Beijing 100101, China; b College of Resources and Environment, University of Chinese Academy of Sciences, Beijing 100049, China

**Keywords:** Land-river-estuary continuum, Carbon neutrality, Carbon source and sink, Carbon transportation, Carbon sedimentation, Carbon cycle

## Abstract

In terrestrial ecosystems, carbon (C) transportation and C pool transformation processes both occur at the land-river-estuary continuum. Moreover, C budget and C balance processes are generally critical in achieving the C neutrality of terrestrial ecosystems. This study analyzes key C transportation processes at multiple interfaces that collectively constitute the land-river-estuary continuum, discusses C transportation and sedimentation processes at the land-river interface, and reveals aquatic plant C sequestration coupling processes and associated productivity. Transformation mechanisms of inorganic-organic C pools are also investigated here as well as a systematic evaluation of C transport flux within the different interfaces that constitute the land-river-estuary continuum. Results show that the net C sink of terrestrial ecosystems was 1.70 Pg C yr^−1^, wherein the gross primary productivity (GPP) of global terrestrial vegetation reached 123 Pg C yr^−1^, while rock weathering also consumed 0.30 Pg C yr^−1^ of atmospheric carbon dioxide (CO_2_). Subsequently, the C transported by the land-river-estuary continuum reached 1.70 Pg C yr^−1^. During this process, 0.20 Pg C is deposited and buried in inland water and 1.00 Pg C escapes from inland water systems each year. Therefore, only 0.85 Pg C is transported to the estuary. Finally, this study clarifies control mechanisms of C transportation and transformation processes at the land-river-estuary continuum. The aim of this study is to provide an important scientific basis for the quantitative analysis of C sources and sinks at the land-river-estuary continuum and C neutrality of the biosphere.

## Graphical abstract

**Figure:**
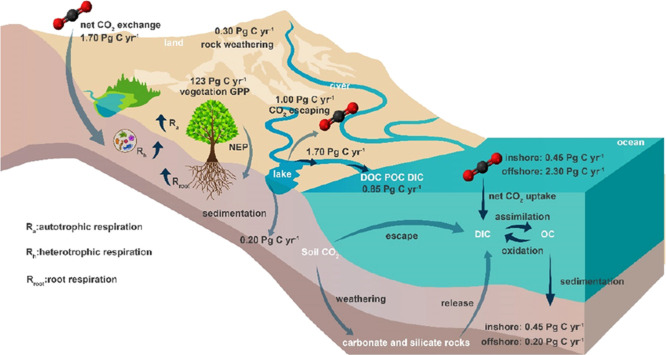

## 1 Introduction

**Fig. 1:**
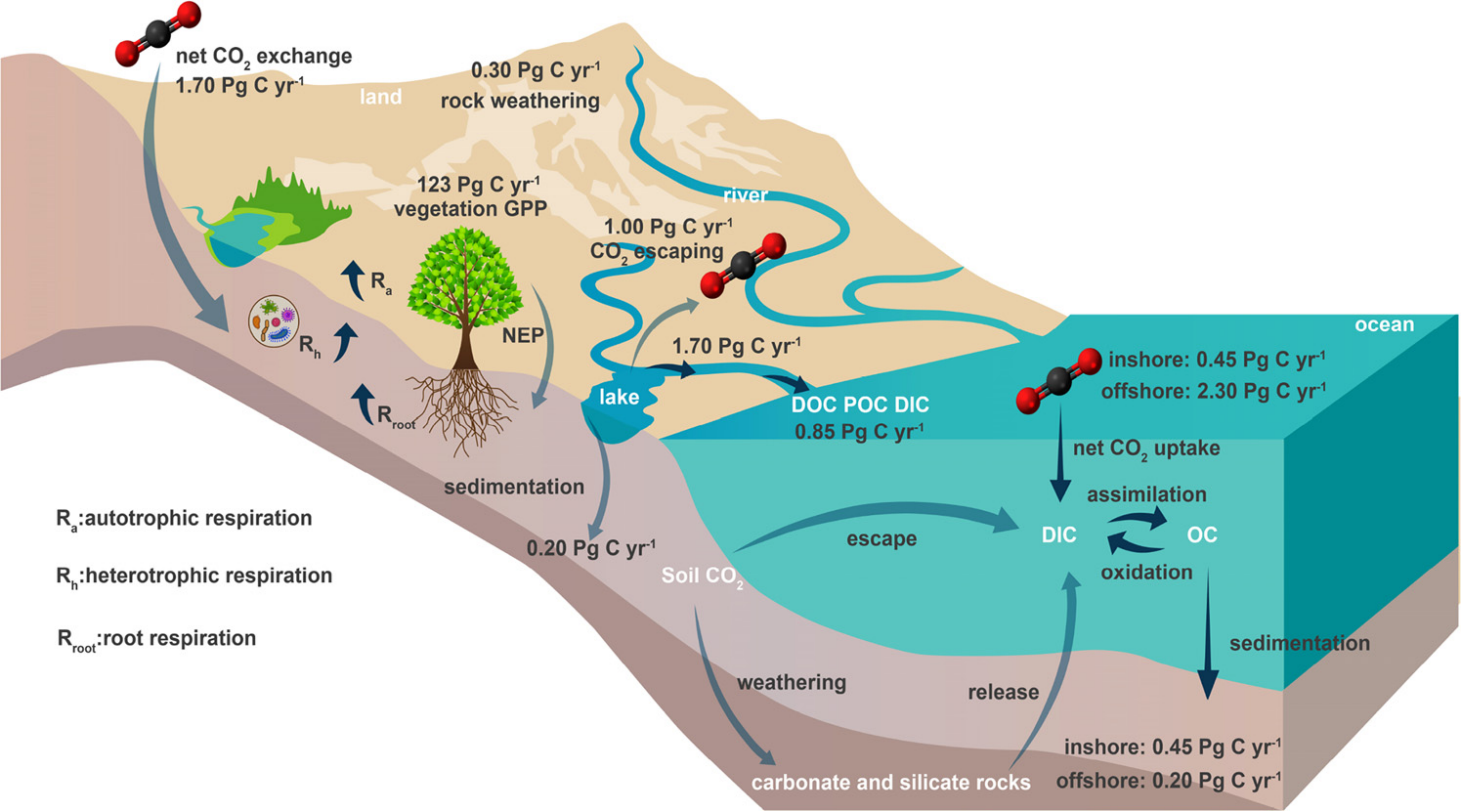
**Carbon transported by terrestrial ecosystem control C escaping from inland water (Table S1 in Supplementary Materials for data source).** CO_2_ exchange, photosynthetic production and respiration of terrestrial vegetation, rock weathering, and sedimentation occur in terrestrial ecosystems. C transports to the ocean through the land-river-estuary continuum in the form of organic carbon and inorganic carbon. C exchange at the air-water interface, transportation, transformation and sedimentation occur in oceanic ecosystems.

Terrestrial and marine ecosystems constitute two of Earth's most important C sinks. They are chiefly connected through riverine transport, which acts as the main channel through which erosive terrestrially derived substances enter into oceanic systems. Material and energy exchanges between the terrestrial biosphere and the lithosphere as well as organic carbon (OC) fixation and oxidation processes together regulate ecosystem C and dioxygen (O_2_) pools. Riverine transport, being the main connective channel of these pools, will act to transport net primary productivity (NPP) (primarily in the form of dissolved organic carbon [DOC] and particulate organic carbon [POC]) from terrestrial to oceanic systems [1]. During transport, part of DOC will rapidly return to the atmosphere through redox reactions, causing “C degassing” to occur between land-atmosphere storage layers [2,3], and the remaining DOC and dissolved inorganic carbon (DIC) are also exported to the ocean [4], [5], [6]. Currently IC and OC export fluxes from global rivers to the ocean reached 0.50–0.70 Pg C yr^−1^ and 0.15–0.35 Pg C yr^−1^ [5]. On the other hand, POC can remain buried in sediment over an extensive period, and the annual global terrestrial to oceanic POC flux has been estimated at $0.20_{-0.07}^{+0.13}$ GgC yr^−1^ [7] (Fig. 1). Therefore, quantitatively estimating the key multi-interfacial C transportation processes and fluxes at the land-river-estuary continuum, and determining whether it is a C source or sink in the land-ocean C transportation processes, is of great significance for accurately quantifying global ecosystem C budget and in-depth understanding of global C cycle and C neutrality.

Inland water systems include rivers, lakes, reservoirs, estuaries, and coastal wetlands, with a combined total surface area of 3.6 × 10^6^ km^2^, accounting for approximately 2.67% of the global area. Lake systems comprise of greater than 90% of inland water bodies, and they are therefore a significant component of the global C cycle [8,9] (Fig. 1). However, a global, standardized, and systematic C source-sink observation system of the land-river(lake-reservoir)-estuary continuum for global inland water systems has yet to be established. Additionally, there remain missing observational data on certain plateau and alkaline lake systems, which causes a significant C flux underestimation at the water-air interface. It is important to note that C budget and C balance processes of the land-river(lake-reservoir)-estuary continuum play a key role in terrestrial ecosystem C neutrality [10,11]. Therefore, it is necessary to establish a comprehensive and standardized scientifically-based C sink observation system of the land-river(lake-reservoir)-estuary continuum to better understand the C source and sink characteristics of inland water bodies and to more accurately assess regulatory mechanisms and pathways to enhance C transportation and C transformation processes of the land-river-estuary continuum.

## 2 Key multi-interfacial carbon transportation processes at the land-river-estuary continuum

**Fig. 2:**
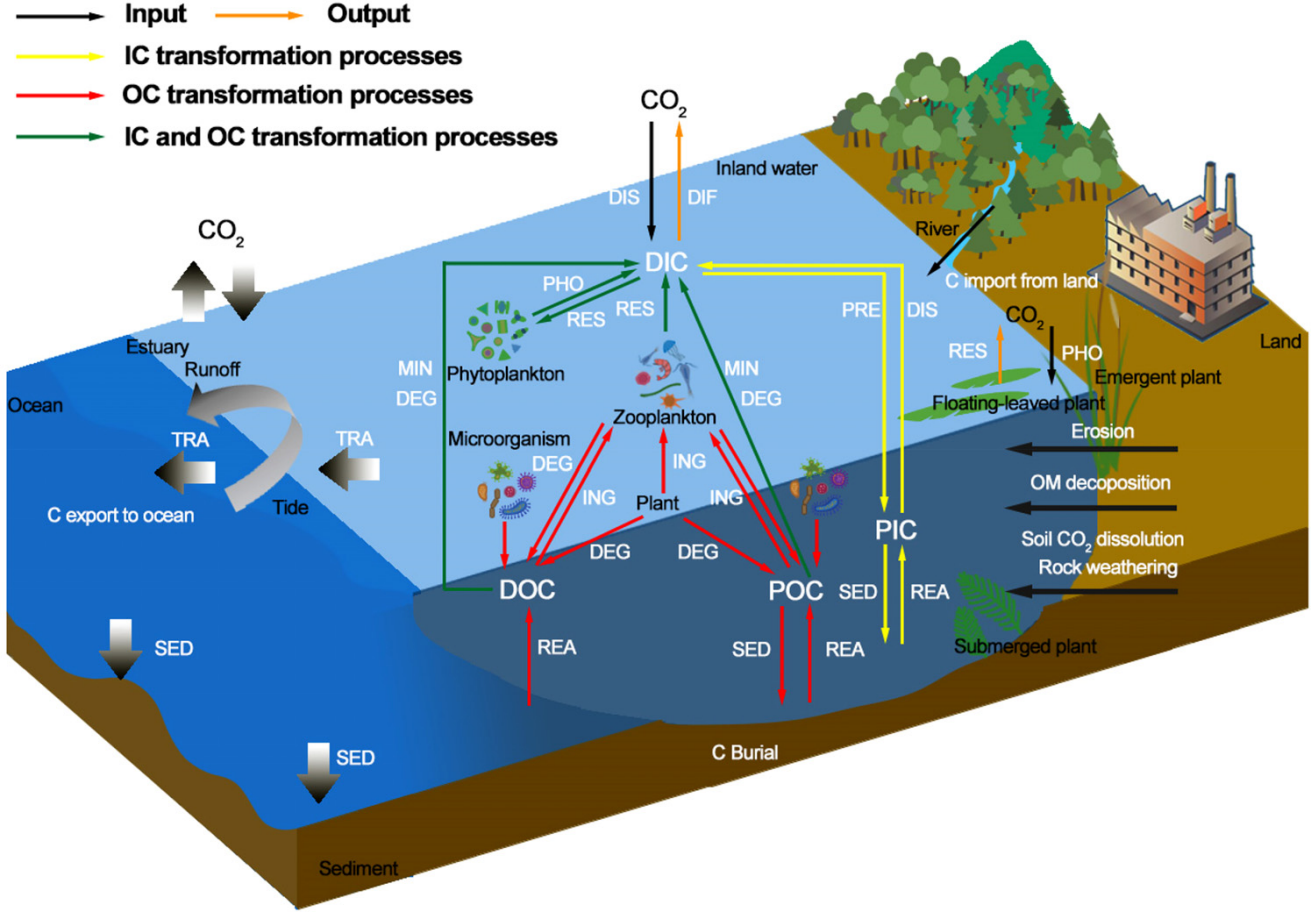
**Carbon transportation and transformation processes at the land-river-estuary continuum.** DIC, dissolved inorganic carbon; DOC, dissolved organic carbon; PIC, particulate inorganic carbon; POC, particulate organic carbon; OM, organic matter; PRE, precipitation; DIS, dissolution; PHO, photosynthesis; RES, respiration; SED, sedimentation; REA, release; MIN, mineralization; DEG, degradation; ING, ingestion; TRA, transportation; DIF, diffusion. Thick arrows indicate carbon processes in the estuary, and thin arrows indicate carbon processes in inland waterbodies including rivers, streams, lakes, and reservoirs.

Terrestrial and atmospheric C are continuously transferred from inland water systems to coastal ecosystems. During this process, C is occasionally deposited in inland waterbodies; some C also returns to the atmosphere in its gaseous forms, while the remaining C is transported to coastal regions via estuaries (Figs. 1 and 2). According to its geographic and geological characteristics, the land-river-estuary continuum can be subdivided into four sectors: upstream, midstream, downstream, and estuary. The upstream sector typically flows through steep plateaus. Although its water volume is low, its gradient is high, resulting in rapid flow velocities and strong erosional forces, being the main sources of suspended material in river systems. The water volume of the midstream sector will gradually increase under a typically progressive gradient, allowing erosion and sedimentation to remain roughly balanced. The downstream sector is characterized by wide river valleys, curved river channels, low flow velocities, high flow volumes, and a considerable sedimentation effect. The estuarine process chiefly acts as a “filter” and “sedimentation tank” [12].

The net C sink of terrestrial ecosystems was 1.70 Pg C yr^−1^, wherein the gross primary productivity (GPP) of global terrestrial vegetation reached 123 Pg C yr^−1^, and rock weathering also consumed 0.30 Pg C yr^−1^ atmospheric CO_2_ [13,14]. Subsequently, C transported by the land-river-estuary continuum reached 1.70 Pg C yr^−1^ [15]. During this process, 0.20 Pg C was deposited and buried in inland water [16] and 1.00 Pg C was escaped from inland water every year, so only 0.85 Pg C was transported to estuary [5]. The offshore water, as a large C sink, can uptake atmospheric CO_2_ by 2.30 Pg yr^−1^ and bury C by 0.20 Pg yr^−1^ [15,17,18]. Due to the differences of estimation methods and the lack of standardized and systematic C source/sink observation system for the land-river-lake system, there exists a great uncertainty in quantifying key multi-interfacial C transportation processes and fluxes at the land-river-estuary continuum, which also leads to the imbalance of C input and output fluxes. Therefore, it is urgent to establish a scientific and systematic C source or sink measurement standard system for accurately quantifying C transportation process fluxes at the land-river (lake-reservoir)-estuary continuum.

### 2.1 Carbon transportation and transformation processes at the land-river interface

Soil biogeochemical processes and solubility characteristics directly affect the quantity and composition of organic and inorganic C components stored in soil that enter into groundwater and river and stream systems [19]. DIC transported from terrestrial inputs into waterbodies mainly derives from the dissolution of soil CO_2_ and the weathering of rock material (i.e., carbonate, silicate, etc.). The CO_2_ in soil mainly derives from biochemical processes, such as plant root respiration, litter decomposition, and microbial respiration, as well as the physical exchange between the soil surface and the atmosphere [20,21] (Fig. 2). DOC transported from terrestrial to river systems and then to groundwater mainly originates from plant sources, such as aboveground litter, belowground litter, and root exudates, as well as through OC decomposition processes in the form of microbial residue and soil organic matter (SOM) [22]. Additionally, soil erosion processes can transport OC from plant sources, microbial residue, SOM, and inorganic carbon (IC), such as through the rock weathering and carbonate mineral products transported to waterbodies in the form of terrestrial POC and particulate inorganic carbon (PIC) [23]. Atmospheric CO_2_ will be directly imported into waterbodies in the form of DIC. Different types of DIC (including aqueous CO_2_ [aq], carbonic acid [H_2_CO_3_], bicarbonate [HCO_3_^−^], and carbonate [CO_3_^2−^]) will either form through dissolution processes at the water-air interface or be fixed into waterbodies through the photosynthesis of floating-leaf and emergent plants [24]. Additionally, different C forms in waterbodies derive from both domestic and industrial wastewater discharge. Groundwater is also a vast C input source of river systems and coastal ecosystems. Nutrient and C concentrations in groundwater can exceed that in surface water; therefore, the transportation of nutrients and C to coastal regions through groundwater may be a greater source compared to that from river systems [25], [26], [27].

### 2.2 Carbon transportation and transformation in inland water systems

#### 2.2.1 Carbon transformation process of the inland water system

The C transformation process of aquatic systems mainly includes those associated with the IC pool, the OC pool, and transformation processes that occur between these two pools at the water-air interface and the water-sediment interface. The IC pool transformation process mainly includes the following: atmospheric CO_2_ forms DIC through dissolution, while aquatic DIC can also be hydrolyzed to release CO_2_ into the atmosphere; DIC and PIC can be transformed through precipitation or dissolution; PIC is ultimately deposited in sediment and can also be released from sediment into waterbodies [28] (Fig. 2). The OC pool transformation process mainly includes the following: plants, animals, and microorganisms in waterbodies are constituents of the OC pool; animals can feed on DOC and POC that derive from both plants and waterbodies; microorganisms can also degrade plant and animal residue; finally, POC can be either buried in sediment through sedimentation processes or released into waterbodies through biogeochemical processes [29]. The transformation process between IC and OC pools mainly includes the following: floating-leaf and emergent plants directly utilize atmospheric CO_2_, while phytoplankton utilizes aqueous CO_2_ (aq) and HCO_3_^−^ in water for photosynthesis from which GPP is produced [30]; floating-leaf and emergent plants release CO_2_ directly into the atmosphere through respiration, while phytoplankton and animals release CO_2_ through respiration, which form the DIC in water [31]. Meanwhile, microbial-induced degradation and mineralization processes in OC pools can also transform OC into IC; DOC in the upper layer of waterbodies can undergo photodegradation under sunlight conditions, producing DIC [32].

#### 2.2.2 Carbon transportation and transformation in river systems

Carbonate and silicate rock weathering processes consume atmospheric CO_2_, which is a net sink of the global carbon cycle [33,34]. Particulate matter produced by rock weathering during river transportation processes also plays a role in C sink processes [34]. Weathered (sediment) particles of silicate and carbonate can interact with DIC in water after flowing into the river systems through runoff [35]. Additionally, the ionic content of river systems closely correlates to the movement of sediment, where they mutually influence each other and are interconnected [36,37]. Sediment minerals convert from one chemical state to another, which is often accompanied by ionic generation and dissolution processes [37]. Erosion, which includes both physical and chemical erosion processes, occurs when water flows through weathered soil. Physical erosion results in sediment particles of differing size, while chemical erosion results in different types of sediment and ions [37]. China is one of the countries with the largest number of river systems in the world, including approximately 50,000 river systems with basin areas greater than 100 km^2^ [38]. However, the total annual sediment transport from China's main river systems was only 340 million tons from 2010 to 2019, which was 77% lower than the multiyear average (1950–2015) [39]. Accordingly, the impact of river C transportation on C cycling processes of terrestrial systems cannot be ignored.

#### 2.2.3 Carbon fixation processes of aquatic plants

**Fig. 3:**
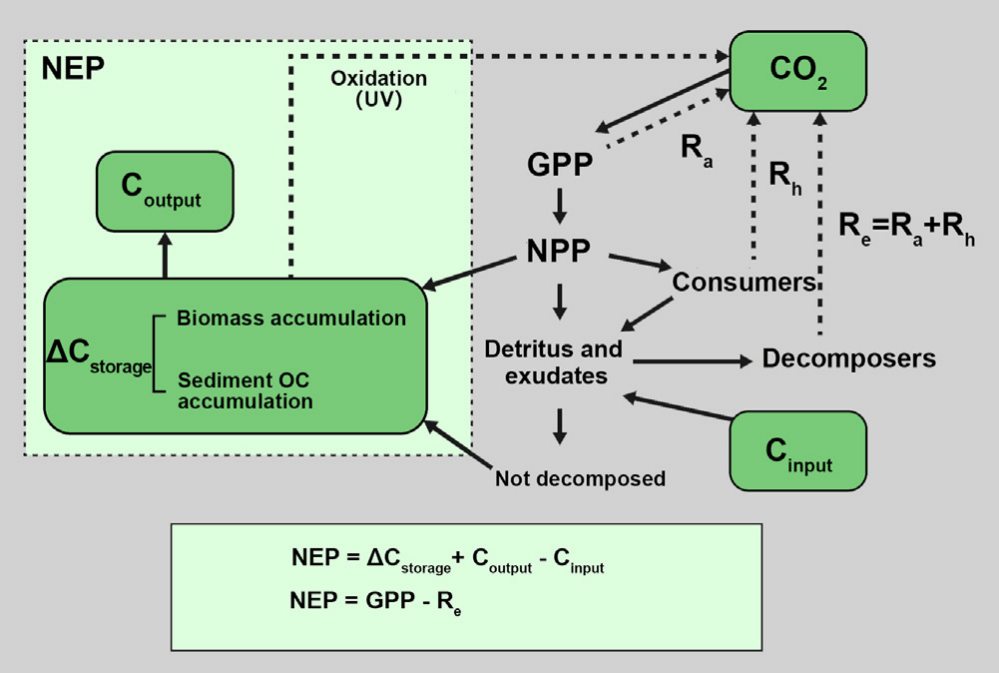
**Carbon fixation by aquatic plants and coupling processes of various trophic levels in inland water (revised from Staehr et al., 2012).** Total ecosystem respiration (R_e_) is the sum of autotrophic respiration (R_a_) and heterotrophic respiration (R_h_). GPP, gross primary productivity; NPP, net primary productivity; NEP, net ecosystem productivity; UV, ultraviolet. Dashed lines indicate carbon mineralization and solid lines indicate carbon assimilation. Compared with net heterotrophic ecosystems (NEP < 0), net autotrophic aquatic ecosystems (NEP > 0) have a net accumulation and/or net export of organic matter.

Aquatic plants absorb C through photosynthesis and then fix it into organic compounds [40] (Fig. 3). Meanwhile, organic matter will return to the atmosphere through plant respiration (autotrophic respiration) and decay in lake water and sediment (heterotrophic respiration), forming the “atmosphere-aquatic plants-river/lake/reservoir-atmosphere” C cycling system [41]. Compared to terrestrial plants, aquatic plants have the capacity to utilize HCO_3_^−^ during photosynthesis. Approximately 50% of submerged plants utilize HCO_3_^−^ as an IC source in addition to CO_2_ [42]. Compared to phytoplankton, the utilization rate of incident light of large aquatic plants is higher in the water column. The productivity per unit biomass or the chlorophyll content of large aquatic plants is also higher, having an overall stronger C fixation capacity. Previous study has shown that the average annual C sequestration capacity of a dense submerged plant community is generally 500–2000 g C m^−2^ yr^−1^, which is much higher compared to phytoplankton (10–200 g C m^−2^ yr^−1^) and greater than many emergent plant communities (500–1000 g C m^−2^ yr^−1^) [43]. 25%-50% of the C in aquatic plants is buried in sediment in the form of cellulose, lignin, and hemicellulose, which are difficult to decompose and are thus not bioavailable, potentially prolonging the C cycling period [44,45].

Additionally, although climate warming will increase OC loss, it will also promote improvements in lake photosynthetic rates and net productivity [16,46]. Large aquatic plants take root at the bottom of waterbodies, creating a diverse sediment-water interface which provides habitat for more aquatic organisms. The diversity of aquatic ecosystems with large aquatic plants is much higher compared to waterbodies dominated by phytoplankton, and the gross production of such ecosystems is very high [41]. Therefore, the net productivity of aquatic lake plants is not only related to the type of aquatic plants and its total biomass, but it is also closely connected to the growth process of aquatic plants as well as external environmental factors, such as temperature and sunlight [47]. Additionally, compared with the microbial C pump process in the ocean [48], the living environment of microorganisms in rivers with strong hydrological fluidities is different from that of oceans, accordingly, a deep research gap on microbial C pump for C sequestration exists in freshwater ecosystems, which can be further studied in the future.

### 2.3 River-estuarine carbon transportation

Estuaries are the transitional zone between river to oceanic systems. In estuaries, terrestrial runoff is mixed with seawater. On the one hand, the hydrodynamic condition of an estuary, which is affected by both tides and runoff, will lead to a short residence time and cycling period as well as a more intense physical circulation of waterbodies [49]. On the other hand, under conditions where freshwater and saltwater mix, the solid-liquid balance in the water of estuaries will alter. Different C forms transported by river systems are prone to flocculation, sedimentation, or adsorption-desorption after entering an estuary, acting as a “filter” and “sedimentation tank” [12]. Biogeochemical processes, such as photosynthesis and respiration, dissolution and precipitation, degradation and mineralization, and sediment release and deposition, occur in estuaries, and DOC will rapidly return to the atmosphere through oxidation processes while POC will be buried in sediment over an extended timespan because of its unique hydrodynamic and geochemical properties [7]. Dynamic environmental conditions along the ever-evolving ecotone provide a rich and unique ecological niche while also providing high biodiversity and productivity development [25]. Consequently, estuaries provide many ecosystem services, such as sediment and C storage, the buffering of storm and flood events, and fishery production [12].

## 3 Controlling mechanisms of carbon transportation and transformation at the land-river-estuary continuum

**Fig. 4:**
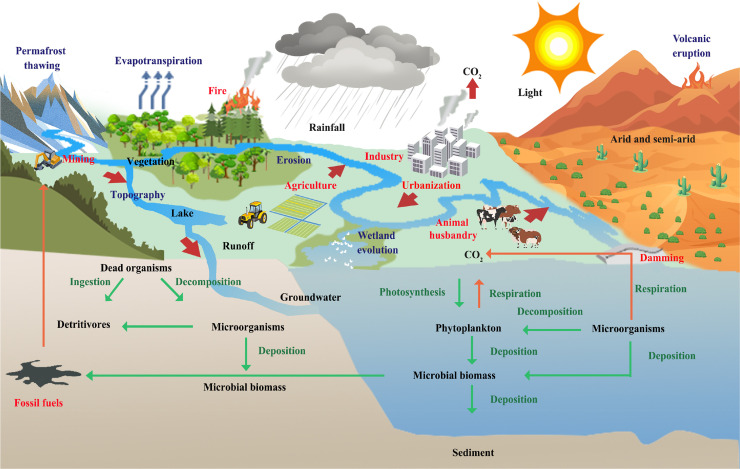
**Control mechanisms of carbon transportation and transformation at the land-river-estuary continuum.** Red words denote anthropogenic activities processes, blue words denote climate change processes; red thick arrows denote carbon transportation and emission processes, green thin arrows denote carbon input and biotransformation processes, and orange thin arrows denote carbon output and biotransformation processes.

Climatic conditions, vegetation coverage, topography, and landform types (and other factors) in watersheds regulate C components and flux rates at the land-water interface by influencing the production and decomposition of SOM, soil erosion, carbonate weathering, and other such processes [23,50] (Fig. 4). Studies have found that China's Yangtze River and Pearl River, which are in the subtropical monsoon region, as well as river DOC flux from these river systems, significantly and positively correlate to terrestrial NPP, while China's Yellow River, which is in arid-semi-arid regions, as well as river DOC flux, is mainly affected by precipitation-based erosion [51]. Physical and chemical factors (i.e., sunlight, temperature, dissolved oxygen, pH, and nutrient content) can affect the photosynthesis of aquatic autotrophs [31,52]. Based on a study on waterbody primary productivity in the Poyang Lake basin, seasonal changes were found to result from the comprehensive regulation of sunlight, pH, and nutrients, of which sunlight was determined to be the main controlling factor [53]. Additionally, increases in surface water temperature and extensions of seasonal stratification have promoted primary productivity of Superior Lake in the United States [54].

The release of CO_2_ at the water-air interface is mainly controlled by the biological pump and the solubility pump of waterbodies. The biological pump mainly relies on photosynthesis, respiration, and microbial degradation, while the solubility pump is mainly affected by factors such as wind speed, water temperature, pH, and carbonate decomposition [18]. Yan et al. [55] found that the average CO_2_ emission flux at the water-air interface of 17 lake systems on the Qinghai–Tibet Plateau was 73.7 (0.9–295.3) mmol m^−2^d^−1^, and associated temporal and spatial changes were mainly significantly related to DOC, dissolved organic nitrogen (DON), salinity, and water temperature, while C emission flux of saline lake systems was higher compared to freshwater lake systems. Additionally, in ten reservoirs representative of the diversity of French hydropower reservoirs, higher fluxes were measured in summer than in spring and winter, which were mainly affected by temperature, organic matter input, vegetation cover in the basin, and water retention time [56]. Erosion and water flow velocities on land directly regulate the C sedimentation of waterbodies. Wang et al. [57] found that the average C deposition rate of lake systems on the Qinghai–Tibet Plateau was 0.4 mm yr^−1^, for which precipitation and the watershed-lake area ratio mainly regulated watershed erosion and flow velocity.

Anthropogenic activities and climate change further cause C balance changes in waterbodies. Such activities include domestic and industrial wastewater discharge, the construction of dams and reservoirs, changes in land-use types, etc. The effects of climate change are mainly observed in rising temperatures and changes in precipitation patterns (i.e., more frequent extreme precipitation events) [8,31,57,54]. Climate warming directly affects the primary productivity as well as the degradation and mineralization process of organic matter in waterbodies. Song et al. [31] found that a 1 °C increase in temperature in river systems throughout the world will reduce NPP by approximately 23.6% and cause a 0.0194 Pg C yr^−1^ increase in river C emissions. Additionally, permafrost thaw, sea level rise, and wetland degradation under climate change conditions will cause changes to both biogeochemical and C cycling processes at the land-river-estuary continuum [58]. Elevated atmospheric CO_2_ concentrations may increase dissolved CO_2_ levels in freshwater systems via water-air exchanges, thereby enhancing primary productivity in inland waters [59]. However, in waters with excess N and phosphorus loads, increased atmospheric CO_2_ may also promote algal growth and increase algal biomass, resulting in water eutrophication, exacerbating OC cycle, deteriorating water quality, and ecosystem imbalances [59,60].

Changes in precipitation patterns, especially extreme weather events (i.e., droughts and floods), will change C outputs at the land-water interface as well as the water retention time, the sedimentation rate, etc. [12]. Studies have found that a single tropical storm can increase riverine DOC concentrations by a magnitude of 330 within a short timespan (i.e., five days), while this output accounts for approximately 43% of the average annual DOC output from river systems [61]. Additionally, high winds and precipitation related to storms will affect changes in physical and chemical environments of global lakes through short-term runoff events from watersheds and physical mixing of the water column, subsequently restructure phytoplankton communities and their dynamics, and lead to changes in ecological functions (e.g., carbon, nutrient and energy cycling) in the short- and long-term [62].

Reservoirs impede waterbody flow velocity in watersheds and reduce their overall carrying capacity. Erosion processes under strong hydro-dynamic conditions will gradually develop into lake sedimentation, and a large amount of C transported by runoff will be deposited into reservoirs [4]. This is conducive to the growth of phytoplankton and aquatic plants [63] while also retarding OC oxidation and decomposition rates [64]. Dams impede C flow along global river networks, leading to enhanced nutrient transformation and elimination, increasing nutrient retention via sedimentation or gaseous elimination in dammed reservoirs, subsequently influencing downstream terrestrial and coastal environments [65].

Many influencing factors of river flow and sediment processes include the construction and scheduling of large-scale water conservancy projects, the implementation of water and soil conservation projects, sediment diversion from irrigation engineering, sediment mining activities in river systems, and climate change, which may produce obvious effects on river sediment flux [66,67]. For example, the construction of large-scale reservoirs along with the Three Gorges Dam as well as the implementation of upstream water and soil conservation projects is the main driving mechanisms for the reduction in sediment flux of the Yangtze River (i.e., the main river outflow to regions around China) [68]. The implementation of soil and water conservation projects in the middle reaches of the Yellow River, characterized by the construction of sediment storage dams and the return of cropland to forestland, is the key driving factor for the rapid decrease in water and sediment flux of this important river system [69]. The decrease in the sediment load of the Pearl River has been affected by the construction of upstream reservoirs and excessive sediment mining activities [66,70]. Furthermore, in a floodplain system along the Kafue River in Zambia, terrestrial particles were retained by the upstream dam and particulate organic matter from algal and microbial sources was released to the river [71]. In the future, under the dual pressure of climate change and anthropogenic activities, changes in river flow and sediment flux are likely to significantly affect river C transportation processes. Therefore, further relevant research is necessary.

## 4 Estimation of carbon transportation flux at the multiple interfaces of the land-river-estuary continuum

**Fig. 5:**
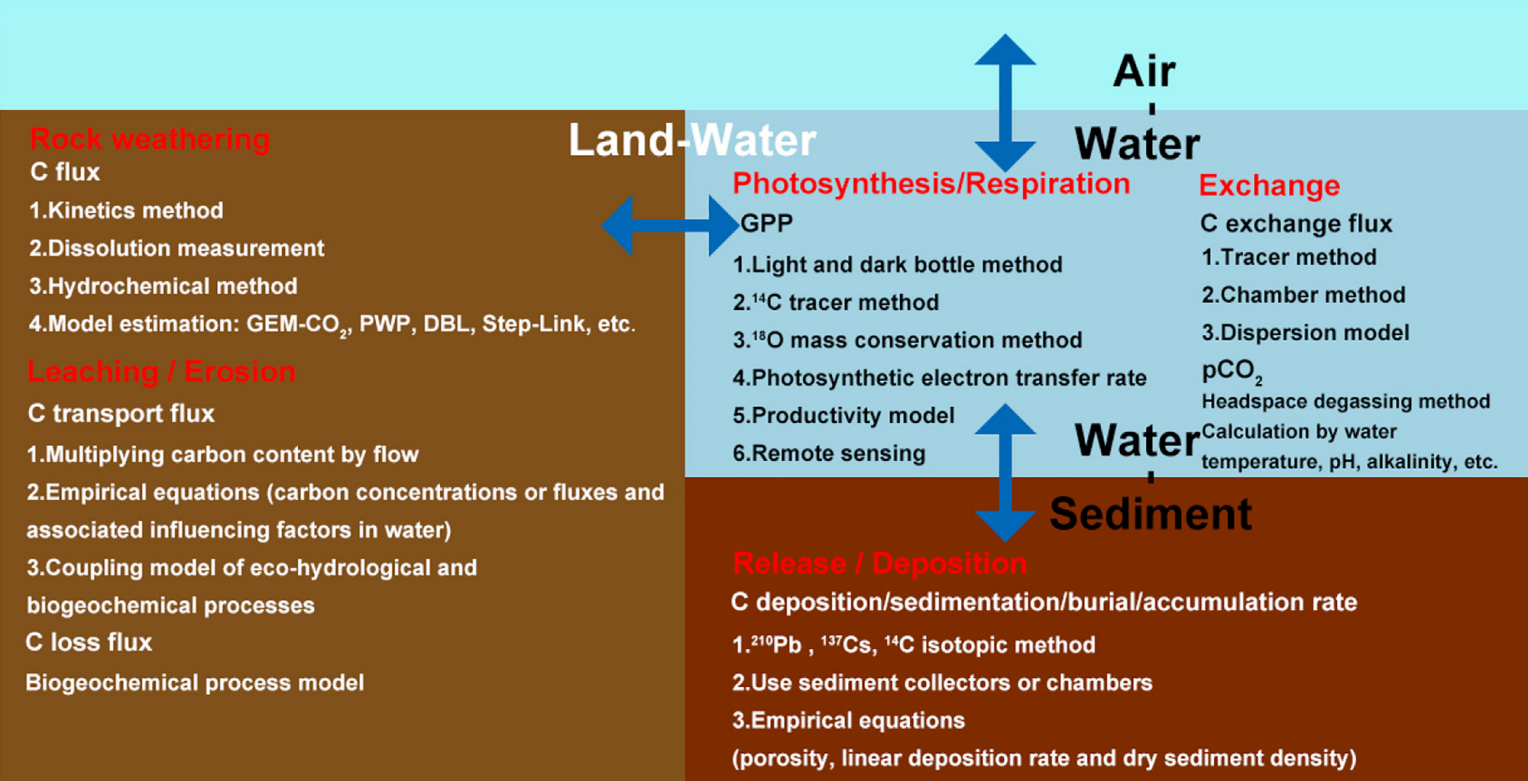
**Estimation methods used to determine key carbon transportation flux at the land-river-estuary continuum.** Rock weathering denotes processes associated with carbon flux by rock weathering. Leaching/Erosion denotes processes associated with carbon flux in river systems. Exchange denotes processes associated with carbon emission flux in inland water systems. Photosynthesis/Respiration denotes processes associated with carbon fixation rate of phytoplankton in inland water systems. Release/Deposition denotes processes associated with carbon sedimentation flux.

Analyzing C flux during leaching and erosion, photosynthesis and respiration, and dissolution and precipitation processes as well as sediment release and sedimentation processes between the land-water, water-air, and water-sediment interfaces are the basis for accurately predicting the impact of environmental change on the C cycling of aquatic ecosystems and understanding associative controlling mechanisms [24,72]. Fig. 5 provides the estimation methods used for C transportation flux at the multiple interfaces of the land-river-estuary continuum.

### 4.1 Carbon flux by rock weathering

Methods used to estimate C sinks caused by rock weathering mainly include the kinetic method, the dissolution measurement method, the hydro-chemical method, model estimations, etc. The kinetic method is based on the relationship between reactant or product concentrations and the reactive times to obtain reactive kinetic parameters, such as the reaction rate constant, activation energy, etc. The reaction rate of a system characterized by these parameters is used to explore reaction mechanisms [73]. It has been both widely and comprehensively studied, leading to a variety of theoretical models, such as the Plummer-Wigley-Parkhurst (PWP) model [74], and the Diffusion Boundary Layer (DBL) model [75]. The dissolution measurement method is used to directly measure the amount of dissolution and establish a dissolution rate model to provide a basis for dissolution rate predictions under different environmental, geological, ecological, climatic (etc.) natural conditions, and to estimate the atmospheric CO_2_ consumed during karstification processes. According to the different dissolution measurement methods available, the dissolution measurement method can be divided into a standard test piece (STP) method [76] and a micro-dissolution method [77]. The hydro-chemical method is based on the fact that the CO_2_ consumed through rock weathering will ultimately be transported from river systems to oceanic systems. Therefore, the total amount of ions transported through river systems is calculated by measuring solute concentrations (i.e., HCO_3_^−^, K^+^, Na^+^, Ca^2+^, and Mg^2+^) in water flux at a basin's outlet and in watershed runoff. Following this, the time it takes for various rock materials to weathering is estimated according to the distribution of rocks in a watershed, and the amount of C consumed by weathering is then estimated [73]. The Global Erosion Model for CO_2_ fluxes (GEM-CO_2_ model) is a successful application of the hydro-chemical method [78].

### 4.2 Carbon flux in river systems

There are three main methods used to estimate C flux in waterbodies transported alongside runoff: 1) through multiplying the C content and flow rate, 2) through constructing empirical equations using C concentration or flux observation data in waterbodies and associated influencing factors, and 3) through coupling ecological hydrological and biogeochemical process models [51,79,80]. The first method (method 1) is limited by a shorter time series or spatial changes in observational data, making it difficult to comprehensively reflect C flux at different temporal or spatial scales [51,81]. Based on empirical equations (method 2), a large amount of measured data is used in its construction, which to a large extent can represent C transportation conditions in different waterbodies [79,82]. The coupling of ecohydrological and biogeochemical process models (method 3) integrates observational data and theoretical models by connecting C processes at the land-water-air interface, which can be used to quantitatively estimate C transportation flux in waterbodies under different climate, land-use, and soil types under real-time dynamics, long-term series, and multiple spatial locations, such as the National Integrated Catchment-based Ecohydrology–Biogeochemical Cycle Model (NICE-BGC model) [80,83].

### 4.3 Carbon emission flux in inland water systems

Through the water-air interface, continuous C exchanges take place between inland waterbodies and the atmosphere. Accurately obtaining temporal and spatial dynamics of C exchange flux at the water-air interface is an important prerequisite for assessing the C sequestration capacity of inland water systems [84]. However, the direct and accurate measurement of CO_2_ flux at the water-air interface is difficult. Quantitative estimation methods that can do so include the following: the tracer method, the chamber method, the dispersion model method, etc. [85,86]. The partial pressure of CO_2_ (pCO_2_) in water is the key parameter to control CO_2_ exchange flux at the water-air interface. The following field sampling methods are used to estimate pCO_2_: (1) direct measurements using the headspace degassing method [87]; (2) indirect estimations using water temperature, pH, alkalinity, and other hydro-chemical indicators [88]. Due to the limitation of observational techniques, relevant studies on C exchange flux in inland water systems have mainly focused on local and regional scales. Moreover, relevant research on the monitoring and evaluation of C exchange flux at the water-air interface has been conducted in several key river, lake, and reservoir systems globally, such as the Yangtze River [89], Taihu Lake [90], and Qinghai–Tibet Plateau lake systems in China [55], some inland lake systems in Africa [91], and lake systems of the Danube Delta in Europe [92]. Given that limited and discrete observed sampling information does not reflect the overall C exchange status of inland waterbodies, a recent study has integrated existing field observational data and combined this data with boundary information from inland waterbodies obtained through remote sensing to achieve an overall estimation of C exchange flux conditions of large-scale inland water systems [93].

Given the obvious temporal and spatial limitation of water sample data collected from different studies during different periods, the C emission estimation methods used for aquatic systems based on the integration of observational data to a certain extent have all neglected to employ temporal and spatial scale characteristics of C exchange variation at the water-air interface. Therefore, there remain considerable uncertainties in the current estimation of total C emissions from inland waterbodies [94,95]. Estimating C emissions from inland water systems must therefore comprehensively consider dynamical spatiotemporal characteristics related to key CO_2_ exchange flux parameters (i.e., pCO_2_) while also developing new methods to estimate spatiotemporal pCO_2_ distribution. This will ensure accurately obtaining dynamical spatiotemporal data of C sources and sinks over an extensive inland water range.

### 4.4 Carbon fixation rate of phytoplankton in inland water systems

Phytoplankton photosynthesis is the main way that C is fixed in waterbodies. GPP directly reflects the C fixation rate of phytoplankton and is also a key indicator for evaluating the C fixation potential of phytoplankton in waterbodies [96]. The current methods used to estimate GPP mainly include the light and dark bottle method, the ^14^C tracer method, the ^18^O mass conservation method, the photosynthetic electron transfer rate method, the productivity model, etc. [18]. Although the light and dark bottle method is a mature and accurate approach, it also has some limitations, such as a prolonged measurement period and cumbersome operation; the ^18^O method and the photosynthetic electron transfer rate method require expensive equipment support, and related technologies are still under development [97]. The productivity model represented by the Vertically Generalized Production Model (VGPM) has been widely used for global water productivity assessments because it comprehensively accounts for innate factors while offering relatively convenient parameter acquisition [98], [99], [100], [101]. For inland water systems, key parameters (i.e., chlorophyll concentration and euphotic depth) must still be obtained through field sampling. Most existing remote sensing inversion models are constructed separately for specific regional waterbodies in combination with measured data, which cannot be used to comprehensively obtain dynamic spatiotemporal information on key parameters for large-scale inland waterbodies [102], [103], [104], [105]. At present, productivity assessments of inland waterbodies in China mainly use field sampling data from specific areas for analysis and research [106,107]. Productivity evaluation research based on remote sensing observations and model simulations is mainly conducted regionally, such as Taihu Lake and the Haihe River [108], [109], [110]. In general, due to the limitation of field sampling and remote sensing inversion techniques, current research on inland water productivity in China is mainly concentrated at local and regional scales, thereby lacking large-scale, long-term, high-precision evaluation results.

### 4.5 Carbon sedimentation flux

Isotopic methods (i.e., ^210^Pb, ^137^Cs, and ^14^C) or sediment collector apparatuses (or other such tools) are mainly used to estimate C sedimentation flux in waterbodies to determine the C deposition/sedimentation/burial/accumulation rate [111]. Besides the isotopic methods, the labelled markers have also been widely used in the wetlands for the sedimentation flux measurements. Some studies have also estimated the C sedimentation rate using porosity and other estimation indexes [46]. It has been estimated that 3.27 Pg C has been buried in lake sediment on the Qinghai–Tibet Plateau over the last 12,000 years, and the average deposition rate is 0.4 mm yr^−1^ [57].

## 5 Prospect

Coupling C exchange and transportation processes at the land-river-estuary continuum play a critical role in global change, the C cycle, and C neutrality of the earth system. River systems are the connection C channels between terrestrial and oceanic systems. Although the amount of C flux from river systems is less than other linked global C cycling interfaces, riverine C flux is unidirectional and its magnitude is the same as the net C flux between the atmosphere-ocean interface as well as that of fossil fuel emissions [12,17,112]. Therefore, greater attention must be paid to C budget and balance processes of the land-river-lake(lake-reservoir)-estuary-ocean continuum. Quantitatively evaluating the C budget and C balance flux of each ecosystem, analyzing the control mechanisms of C cycling, and resolving the issue of the “C source and sink” at the land-river-estuary continuum can provide a theoretical and practical scientific basis for C neutrality of the global ecosystem.

The land-river-estuary continuum inputs terrestrial OC and IC into rivers, which are ultimately transported to estuaries through diffusion and dissolution in rivers/lakes/reservoirs, biological C fixation and decomposition, and sedimentation burial [12,23,28]. Biophysical and chemical interactions associated with these processes are very complex, so it is difficult to analyze key multi-interfacial C transportation processes and their mechanisms at the land-river-estuary continuum at regional and global scales. Additionally, the effects of climate change and anthropogenic activities on some C transportation processes at the land-river-estuary continuum have been identified at regional and global scales [53,62,65]; however, it is challenging to accurately quantify the degree of influence of these changing environmental factors on C fluxes. Under the background of future climate change and anthropogenic activities, changes in river water and sediment fluxes, C sequestration by submerged plants, and accurate quantification of exchange fluxes at the water-air interface are all worthy of further research. This study found that C fluxes (C sink) from deposition and transportation at the land-river-estuary continuum were greater than C fluxes (C source) from emissions to the atmosphere [5,15,16], which indicates that integration of multiple studies and differences in estimation methods result in great uncertainties in quantification of key multi-interfacial C transportation processes and fluxes at the land-river-estuary continuum. To support relevant C neutralization policies, it is essential to integrate the spatiotemporal evolution and quantification of key multi-interfacial C transportation processes and their fluxes at the land-river-estuary continuum in the earth system model.

In recent years, many relevant studies on lake and other freshwater C cycling processes have been published in authoritative journals such as *Nature, PNAS, Nature Geoscience*, and *Ecology Letters* [8,91,[113], [114], [115]]. However, these studies were based on conventional large-scale field sampling methods in a variety of different regions [91,113]. Conventional research methods such as sampling and analysis are time-consuming and laborious. Owing to the high temporal and spatial heterogeneity of inland water systems, the limited available data do not adequately represent all waterbody types and their associated ecosystems, which will lead to significant errors in estimations [8,115,116]. The rapid, large-scale, and periodic characteristics that the satellite-based remote sensing approach offers is a far better method to investigate ecological C cycling processes, such as river, lake, and reservoir systems. This is particularly the case for satellites that incorporate water-based spectral bands suitable for inland water systems (i.e., Sentinel-3 OLCI, the European Space Agency [ESA], and China's high-resolution remote sensing satellite network). This approach can provide important technical support for C cycling research of river-lake-reservoir ecosystems based on the satellite remote sensing technique [11]. Therefore, it is critical to establish a comprehensive and standardized scientifically-based measurement system for inland water systems, which can be used to more accurately quantify the “C source and sink” of the land-river-estuary continuum. This will also be of great significance for determining C budget and C balance in the global terrestrial ecosystem and achieving C neutrality.

## Declaration of competing interest

The authors declare that they have no conflicts of interest in this work.

## Appendix Supplementary materials

Image, application 1
