# Supplementary material for: Carbon transportation, transformation, and sedimentation processes at the land-river-estuary continuum
Source: Fundam Res. 2022 Jul 28;4(6):1594–602. doi: 10.1016/j.fmre.2022.07.007 (PMC11670669; doi:10.1016/j.fmre.2022.07.007)
Supplement: Supplementary file 1 [file mmc1.docx]

**Title：Carbon transportation, transformation, and sedimentation processes at the land-river-estuary continuum**

**Table S1 Data source in Fig.1.**

| Data | C flux (Pg C yr^-1^) | Data Reference |
| --- | --- | --- |
| Vegetation net carbon exchange flux | 1.70 | [1, 2] |
| Vegetation GPP | 123 | [1, 2] |
| Rock weathering carbon flux | 0.30 | [1, 2] |
| Inland waterbody net carbon emission flux | 1.00 | [3] |
| Inland water sediment carbon burial flux | 0.20 | [4] |
| Riverine carbon transportation flux to lake | 1.70 | [3] |
| Riverine carbon transportation flux to ocean | 0.85 | [5] |
| Riverine POC transportation flux to ocean | 0.20 (Gg C yr^-1^) | [6] |
| Coastal net carbon uptake and burial flux | 0.45 | [5] |
| Marine net carbon uptake flux | 2.30 | [7, 8] |
| Marine carbon burial flux | 0.20 | [3] |

**References:**

[1] G.R. Yu, X.M. Sun, Principles of Flux Measurement in Terrestrial Ecosystems (Second Edition). Higher Education Press,(Beijing, 2017).

[2] G.R. Yu, X.Q. Zhao, G.H. Liu, Analysis on technical approaches and potential of increasing carbon sink in China's terrestrial ecosystem, Science Press,(Beijing, 2018).

[3] P. Ciais, et al., Carbon and Other Biogeochemical Cycles, Cambridge University Press,(Cambridge, United Kingdom and New York, NY, USA., 2013).

[4] R. Mendonça, et al., Organic carbon burial in global lakes and reservoirs, Nature Communications 8(1) (2017) 1694.

[5] J.E. Bauer, et al., The changing carbon cycle of the coastal ocean, Nature 504(7478) (2013) 61-70.

[6] V. Galy, B. Peucker-Ehrenbrink, T. Eglinton, Global carbon export from the terrestrial biosphere controlled by erosion, Nature 521 (2015) 204-207.

[7] Y. Gao, T.T. Yang, Y.F. Wang, G.R. Yu, Fate of river- transported carbon in China: implications for carbon cycling in coastal ecosystems, Ecosystem Health and Sustainability 3(3) (2017) e01265.

[8] Y. Gao, et al., Determining dominating control mechanisms of inland water carbon cycling processes and associated gross primary productivity on regional and global scales, Earth-Sci. Rev. 213 (2021) 103497.
